# Dynamic Generation of Spatially Variable Vector Beams Using Cascaded Moiré Metasurfaces

**DOI:** 10.1002/nap2.70261

**Published:** 2026-08-25

**Authors:** Siyu Guo, Zhongzhu Liang, Zhe Wu, Xintong Wei, Shijia Zhu, Junying Liu, Siyu Liu, Qiaohan Liu, Xiaoyan Shi, Fuming Yang, Haiyang Xu

**Affiliations:** ^1^ State Key Laboratory of Integrated Optoelectronics and Key Laboratory of Ultraviolet Light‐Emitting Materials and Technology of Ministry of Education College of Physics Northeast Normal University Changchun China

**Keywords:** dynamic regulation, metasurfaces, moiré effect, vector beams

## Abstract

Vector beams with spatially inhomogeneous polarization distributions have demonstrated potential in applications such as optical micromanipulation, high‐resolution imaging, and high‐capacity optical communications. Metasurfaces have emerged as a compact platform for generating vector beams. However, achieving continuous dynamic adjustment of polarization order and spatial position within the same device remains a challenge. This research proposes and validates a mechanism for multidimensional dynamic light field control based on cascaded metasurfaces. In this approach, the metasurface employs phase modulation and the Moiré effect to generate conjugate vortex beams under circularly polarized incident light. Under linearly polarized illumination, coherent superposition generates vector beams with tunable polarization order and controllable focal position. Different rotation parameters govern the polarization order and azimuthal focal position, with limited cross‐influence over the investigated range. For polarization orders of 0–6 and lateral offsets up to 30μm, the main polarization topology is preserved and all target OAM purities exceed 75%. At larger offsets, aberrations reduce the purity, although the target mode remains dominant and the focusing efficiency stays above 67%. This research expands the degrees of freedom for high‐precision reconstruction of complex dynamic light fields, providing support for optical applications such as multi‐target optical trapping and dynamic light routing.

## Graphical abstract

A three‐layer cascaded moiré metasurface dynamically generates vector beams with tunable polarization order and controllable lateral focal positions. Relative rotation of the first two layers adjusts the conjugate OAM components that form the vector beam, whereas the third layer steers its focus across the transverse plane, preserving the dominant OAM content and polarization topology within the investigated operating range.

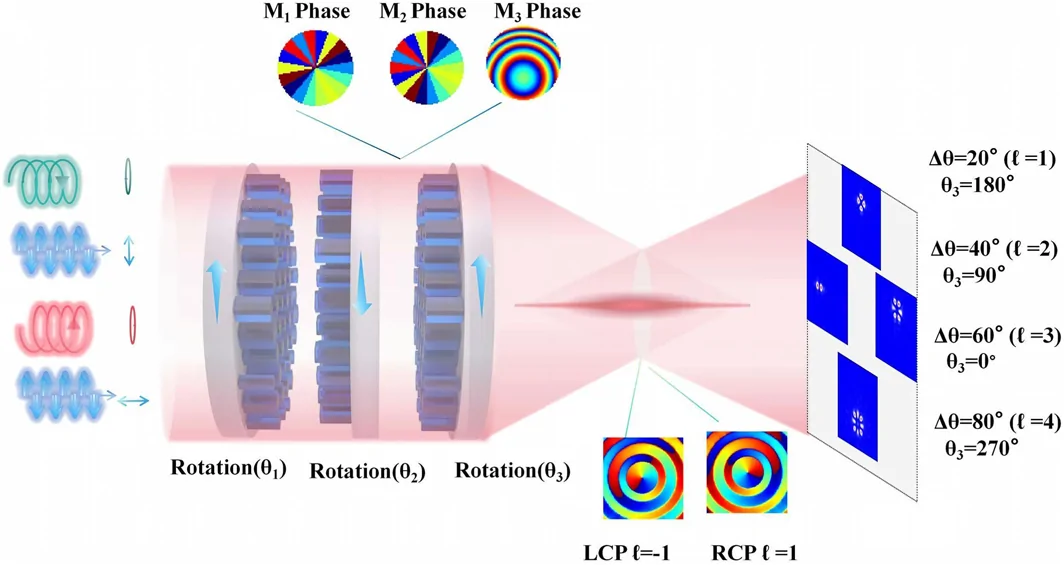

## 1 Introduction

Polarization is a fundamental property of light that plays a crucial role in its interaction with matter. A vector beam (VB) exhibits a spatially inhomogeneous distribution of polarization states across its cross‐section, in contrast to spatially homogeneous polarized light, such as linearly or circularly polarized light. Vector beams are highly attractive for numerous advanced applications, including optical particle trapping (optical tweezers) [1, 2, 3], super‐resolution imaging beyond the diffraction limit [4, 5], and high‐capacity optical communications [6, 7]. Traditionally, spatial light modulators (SLMs) [8, 9] or cascaded refractive/diffractive optical elements, such as q‐plates [10, 11], have been employed to generate and control vector beams. However, these conventional devices are typically large, inefficient, and often result in highly complex systems, making it challenging to meet the demands of modern integrated optics for dynamic multi‐degree‐of‐freedom light field manipulation.

Metasurfaces, as two‐dimensional planar devices composed of subwavelength artificial microstructures, have emerged as a new platform for generating and manipulating vector light beams [12, 13, 14, 15, 16] due to their powerful and flexible ability to control the amplitude [17, 18, 19, 20], phase [21, 22, 23, 24, 25], and polarization [23, 26, 27, 28, 29] of light waves. Cui et al. [13] utilized longitudinal multiplexing to generate vector beams with fixed polarization orders at different axial positions, thereby enabling the construction of three‐dimensional vector light fields. He et al. [14] employed wavelength multiplexing to combine topological charge control with wavelength degrees of freedom, allowing different wavelengths to carry distinct topological charges. Wang et al. [30], through polarization multiplexing, generated vector vortex beams with opposite topological charges in orthogonal circularly polarized channels. However, most of these studies lack the capability for continuous and dynamic control of the light field.

Cascaded metasurfaces based on the moiré effect can tune the composite phase by changing the relative rotation angle between layers, providing an effective route for dynamic light‐field control [28, 29, 30, 31]. Previous studies have demonstrated dynamic tuning of vector‐beam polarization order [31], vortex‐beam topological charge [32, 33], and focal length [34] or focal position [35]. However, several optical‐field parameters are governed by the same phase profile or external control variable, complicating the relationship between the control input and the resulting field. Applications such as three‐dimensional optical manipulation, free‐space beam tracking, and dynamic optical routing require control over both the beam mode and its spatial position. Therefore, it is desirable to assign the polarization order and lateral focal position to different control parameters while preserving the selected modal characteristics during spatial addressing. Dynamic joint control of these two degrees of freedom remains an area for further exploration.

To overcome the challenges of multidimensional dynamic manipulation, this study presents a three‐layer cascaded metasurface architecture that enables continuous control over both the lateral focusing position and the polarization order of a vector beam by exploiting the dynamic tunability of the moiré effect. The first two layers of the metasurface (M_1_ and M_2_) consist of anisotropic silicon nanowaveplates that operate via geometric phase modulation. By rotating these two layers relative to each other, the moiré effect dynamically generates coaxial vector beams with continuously tunable polarization orders under linearly polarized incident light, effectively converting mechanical rotation angles into continuously variable conjugate vortex phases This study introduces a third metasurface, M_3_, composed of isotropic square silicon nanopillars. By employing a polarization‐insensitive propagation phase, M_3_ enables off‐axis focusing, whereas its rotation controls the azimuthal focal position. Consequently, the polarization order and lateral focal position are governed by the relative rotation of M_1_ and M_2_, and the rotation of M_3_, respectively. Simulations at 10.6 μm demonstrate that, for polarization orders ranging from 0 to 6 and lateral offsets up to 30 μm, the primary polarization topology is preserved, and all target orbital angular momentum (OAM) mode purities remain above 75%. At larger offsets, off‐axis aberrations reduce mode purity, although the focusing efficiency remains above 67%. These results demonstrate approximately independent control of polarization order and lateral focal position within the investigated range, providing an integrated approach for multi‐target optical manipulation and dynamic optical routing.

## 2 Principle

An optical vortex is characterized by an azimuthal phase factor exp(ilα), where l denotes the topological charge [36]. Following the complementary angular‐phase construction of rotational moiré metasurfaces [34, 35], M_1_ and M_2_ are assigned mutually conjugate quadratic azimuthal phase profiles.

(1) $$\phi_{1}\left(\alpha \right)=\beta \alpha^{2},$$

(2) $$\phi_{2}\left(\alpha \right)=-\beta \alpha^{2},$$

here, *β* is the adjustment coefficient, and *α* is the azimuthal angle. When these two layers of metasurfaces undergo mechanical rotations of *α*_0_ and ‐*α*_0_ around the optical axis, respectively, the total moiré phase can be expressed as follows:

(3) $$\phi_{12}\left(\alpha \right)=\beta {\left(\alpha +\alpha_{0}\right)}^{2}-\beta {\left(\alpha -\alpha_{0}\right)}^{2}=4\beta \alpha_{0}\alpha .$$

This corresponds to a vortex phase that varies linearly with the azimuthal angle.

To accurately map the theoretical moiré model onto a physical device, we discretize the metasurface in‐plane into N quantized sectors. As the relative rotation about the central optical axis occurs, the continuous spatial azimuth is mapped onto discrete integer orders. We assigned the structural orientation angles of M_1_ and M_2_ to parabolic discrete quantization distributions with the same sign, and their corresponding total phases are defined as follows [31, 36]:

(4) $$\phi_{1}^{OAM}=-\frac{m_{1}^{2}}{N}\pi -n\frac{m_{1}}{N}\pi ,$$

(5) $$\phi_{2}^{OAM}=-\frac{m_{2}^{2}}{N}\pi -n\frac{m_{2}}{N}\pi ,$$

Here, *m*_1_ and *m*_2_ represent the respective quantization orders, $m_{1}=\text{round}\left(\frac{\alpha +\alpha_{0}}{\Delta \alpha }\right)m_{2}=\text{round}\left(\frac{\alpha -\alpha_{0}}{\Delta \alpha }\right)$ N denotes the total number of quantizations, $N=\frac{2\pi }{\Delta \alpha }$ Δα is the difference in rotation angle between two metasurfaces. *n* is an integer coefficient representing the number of terms required to compensate for the additional geometric phase. *n* = 4 is adopted, and the mechanical rotation step is set to Δ*α* = 20°.

To realize the moiré model described above, M_1_ and M_2_ were constructed using anisotropic rectangular nanocolumns. The unit‐cell geometry is shown in Figure 1a. As shown in Figure 1c, we selected 16 unit cells with a phase difference of π in both the X and Y directions to serve as half‐wave plates. Figure 1d demonstrates that these 16 unit cells exhibit polarization conversion efficiencies above 90%. For an anisotropic meta‐atom with orientation angle θ, operating as a half‐wave plate, polarization conversion introduces a spin‐dependent Pancharatnam–Berry geometric phase [36, 37]:

(6) $$\phi_{PB}^{\sigma }=2\sigma \theta \;\sigma =\pm 1.$$

**FIGURE 1:**
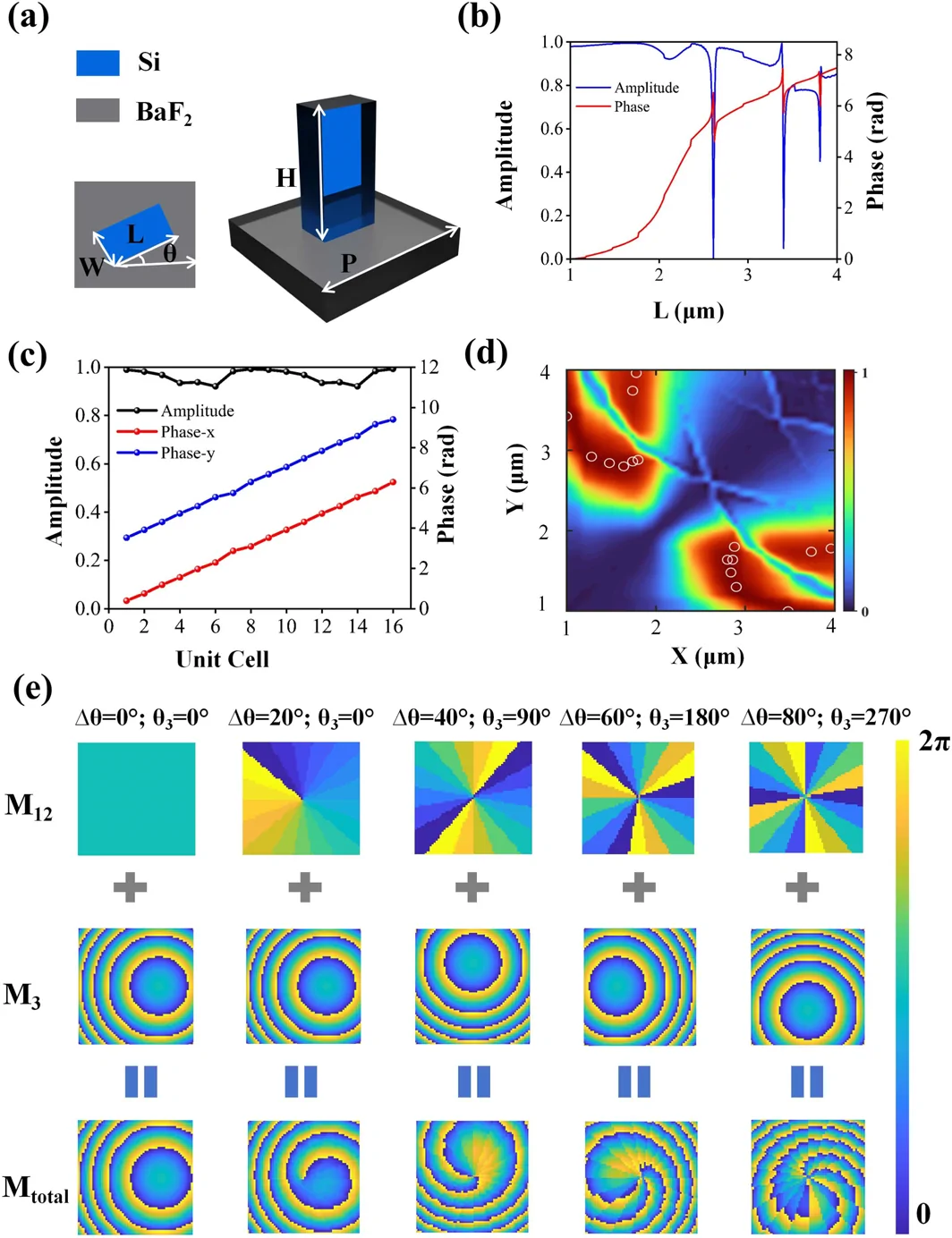
(a) Schematic of the unit‐cell structure. (b) Scanned phase and transmittance of the polarization‐insensitive square unit cells. (c) Phase responses in the x and y directions and transmittance of the 16 selected unit cells. (d) Polarization conversion efficiency of the anisotropic unit cells with different in‐plane dimensions. (e) Phase distributions of the three metasurfaces.

When a beam of left‐circularly polarized light (LCP) and right‐circularly polarized light (RCP) continuously passes through two layers of half‐wave plates, the polarization chirality flips between the layers. Therefore, the total phase of the complete two‐layer cascade is as follows [37, 38]:

(7) $$\phi_{c\text{ascade}}^{LCP}=\phi_{1}^{OAM}-\phi_{2}^{OAM}.$$

(8) $$\phi_{c\text{ascade}}^{RCP}=-\phi_{1}^{OAM}+\phi_{2}^{OAM}.$$

Under linearly polarized light excitation, due to the spin‐flipping property of the Pancharatnam–Berry (PB) phase, the LCP and RCP channels inevitably acquire dynamically tunable topological charges of equal magnitude but opposite signs, *ℓ* and −*ℓ*.

This study introduces a third‐layer metasurface M_3_. M_3_ exhibits an identical phase response for both LCP and RCP channels to avoid disrupting the conjugate topological states generated by the first two layers. Therefore, M_3_ employs an isotropic square silicon nanocolumn structure designed based on the propagation phase mechanism.

The phase profile required to focus the transmitted field at an off‐axis position (*d*_*x*_, *d*_*y*_, *f*) is described by the hyperboloidal metalens phase [39, 40].

(9) $$\phi_{3}\left(x,y\right)=-\frac{2\pi }{\lambda }\left(\sqrt{{\left(x-dx\right)}^{2}+{\left(y-dy\right)}^{2}+f^{2}}-f\right),$$

where *λ* is the operating wavelength, and *f* is the design focal length of the focusing lens. (dx, dy) represents the lateral spatial coordinate of the focal point's deviation from the optical axis on the focal plane. When M_3_ rotates by an angle of *θ*_3_ relative to the entire system, the focal coordinates undergo a strict circular mapping transformation [40]:

(10) $$dx=d_{\text{offset}}\cos\;\theta_{3}.$$

(11) $$dy=d_{\text{offset}}\sin\;\theta_{3}.$$

The radial focal offset is defined as $d_{\text{offset}}=\sqrt{d_{x}^{2}+d_{y}^{2}}$, that is, the distance from the focal point to the optical axis.

Because the transmission phase is completely insensitive to polarization states, the uniaxial rotation of M_3_ is converted into a two‐dimensional translational shift of the focal trajectory, without disturbing the polarization topology of the electric field generated by the first two layers.

Based on the aforementioned layered modulation mechanism, when LCP and RCP light pass through the complete three‐layer cascaded system, their transmitted wavefronts can be rigorously expressed as the linear superposition of the two‐layer moiré topological phase and the M_3_ off‐axis focusing phase.

(12) $$\Phi_{\text{total}}^{\text{LCP}}=-l\alpha +\phi_{3}\left(x,y\right)$$

(13) $$\Phi_{\text{total}}^{\text{RCP}}=l\alpha +\phi_{3}\left(x,y\right)$$

After three‐layer modulation, the LCP and RCP channels each carry vortical topological phases with opposite signs but have the same spatial focusing phase.

In the circular‐polarization Jones basis, a vector vortex beam can be expressed as the coherent superposition of two orthogonally polarized components carrying opposite OAM states [41]:

(14) $${\hat{e}}_{L}=\frac{1}{\sqrt{2}}\left[\begin{array}{l} 1\\ i \end{array}\right].$$

(15) $${\hat{e}}_{R}=\frac{1}{\sqrt{2}}\left[\begin{array}{l} 1\\ -i \end{array}\right].$$

Any linearly polarized (LP) light can be equivalently decomposed into a superposition of LCP and RCP basis vectors with equal amplitude. Therefore, when a linearly polarized beam is incident on this three‐layer system, the transmitted total light field is the linear combination of these two orthogonal channels [41]:

(16) $$E_{\text{out}}\left(\alpha \right)=\frac{1}{\sqrt{2}}\left\{e^{i\left(\Phi_{\text{total}}^{\text{LCP}}+\delta \right)}{\hat{e}}_{L}+e^{i\left(\Phi_{\text{total}}^{\text{RCP}}+\delta \right)}{\hat{e}}_{R}\right\}.$$

The initial phase difference factor, determined by the incident polarization state, is denoted as *δ*. By substituting the total phase and extracting the common envelope term, the Jones vector at the focal plane can ultimately be simplified to the standard form:

(17) $$E_{\text{out}}\left(\alpha \right)=\Psi \left(x,y,z\right)\left[\begin{array}{l} \mathrm{Cos}\left(l\alpha +\delta \right)\\ \mathrm{Sin}\left(l\alpha +\delta \right) \end{array}\right],$$

The spatial complex amplitude envelope, which includes the off‐axis focusing phase, is denoted as $\Psi \left(x,y,z\right)=A_{0}e^{i\phi_{3}\left(x,y\right)}$. In the ideal phase design, the relative rotation of M_1_ and M_2_ primarily alters the polarization order of the vector beam, whereas the rotation of M_3_ mainly adjusts the overall lateral focal position. However, because a practical device is influenced by factors such as finite aperture, phase discretization, interlayer propagation, and off‐axis aberrations, these two control processes are not strictly independent and exhibit limited cross‐influence within the investigated operating range.

This study presents numerical simulations of a cascaded moiré metasurface using the finite‐difference time‐domain (FDTD) method. As shown in Figure 2, the complete cascaded system consists of three metasurface layers, each with a 120 × 120 pixel array, and the focal length is set to 500 μm. The device is designed for an incident wavelength of 10.6 μm. The metasurface consists of silicon [42] nanocolumns positioned on BaF_2_ [43] substrates, which exhibit excellent mid‐infrared transmission properties. Each nanocolumn has a height of 6.5 μm, and the unit cell period is set to 5 μm. For layers M_1_ and M_2_, rectangular nanocolumns measuring 2.84 μm × 1.48 μm were selected to serve as half‐wave plates (HWPs), covering a 2π phase range to achieve geometric phase modulation. For the M_3_ layer, as shown in Figure 1b, 16 isotropic square nanopillars were designed to modulate the propagation phase from 0 to 2π, ensuring that the phase accumulation for orthogonal polarization states is identical and that the overall transmittance remains very high. Detailed structural parameters are provided in Supporting Information S1: Table 1. Figure 1(e) illustrates the theoretical phase distribution and modulation patterns of the cascaded metasurface under different combinations of rotation angles. M_12_, M_3_, and Mtotal represent the moiré superposition phase of the first two layers, the off‐axis focusing phase of the third layer, and the total transmission phase under LCP incidence, respectively. The vortex topological charge undergoes a step change each time Δθ changes by 20°. By changing the relative rotation angle Δθ = *θ*_1_ − *θ*_2_ of M_1_ and M_2_ and the rotation angle *θ*_3_ of M_3_, the topological charge and lateral focal position of the vortex beam can be tuned separately. In the ideal phase model, the two adjustments are determined by different rotation parameters; in the practical cascaded structure, they exhibit only limited cross‐influence over the investigated parameter range.

**FIGURE 2:**
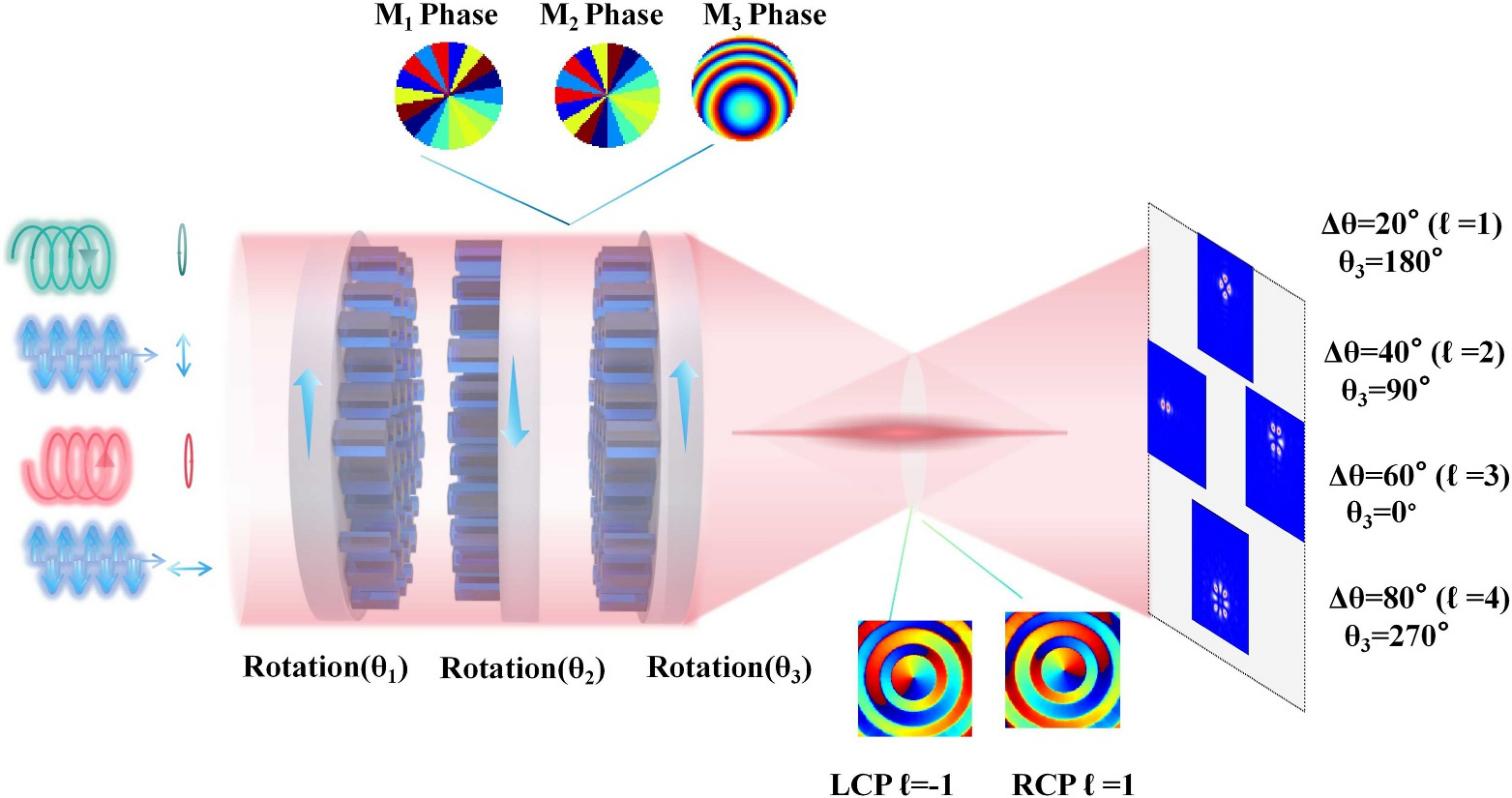
Schematic of the three‐layer cascaded metasurface for dynamic multidimensional light field manipulation. The relative rotation between M_1_ and M_2_ enables orthogonally circularly polarized incident light to generate vortex light fields with opposite signs and continuously tunable topological charges. The uniaxial rotation of M_3_ further achieves transverse focal point shifting. Under linearly polarized excitation, the cooperative rotation of the three metasurface layers dynamically generates high‐order vector beams with independently adjustable polarization orders and spatial positions.

To evaluate the choice of the M_1_–M_2_ separation and its axial tolerance, the interlayer spacing *d* was varied from 0 to 30 μm in 5 μm increments, whereas all other parameters were held constant. As shown in Figure 3g, at *d* = 0 μm, the transmittance, polarization conversion efficiency, and OAM mode purity are 66.07%, 90.78%, and 86.04%, respectively, indicating degraded optical performance at zero separation. At *d* = 5 μm, the transmittance and OAM purity increase to 80.65% and 94.62%, respectively. At *d* = 10 μm, the polarization conversion efficiency reaches 97.98%, whereas the transmittance and OAM purity remain at 80.14% and 93.82%, respectively. Considering optical performance, device compactness, and assembly tolerance, 10 μm was selected. Over the range *d* = 5–30 μm, the conversion efficiency remains above 97.63%, and the OAM purity stays above 92.37%, with variations of only 0.35 and 2.25% points, respectively. Thus, 10 μm lies within a broad stable range rather than representing a narrow optimum.

**FIGURE 3:**
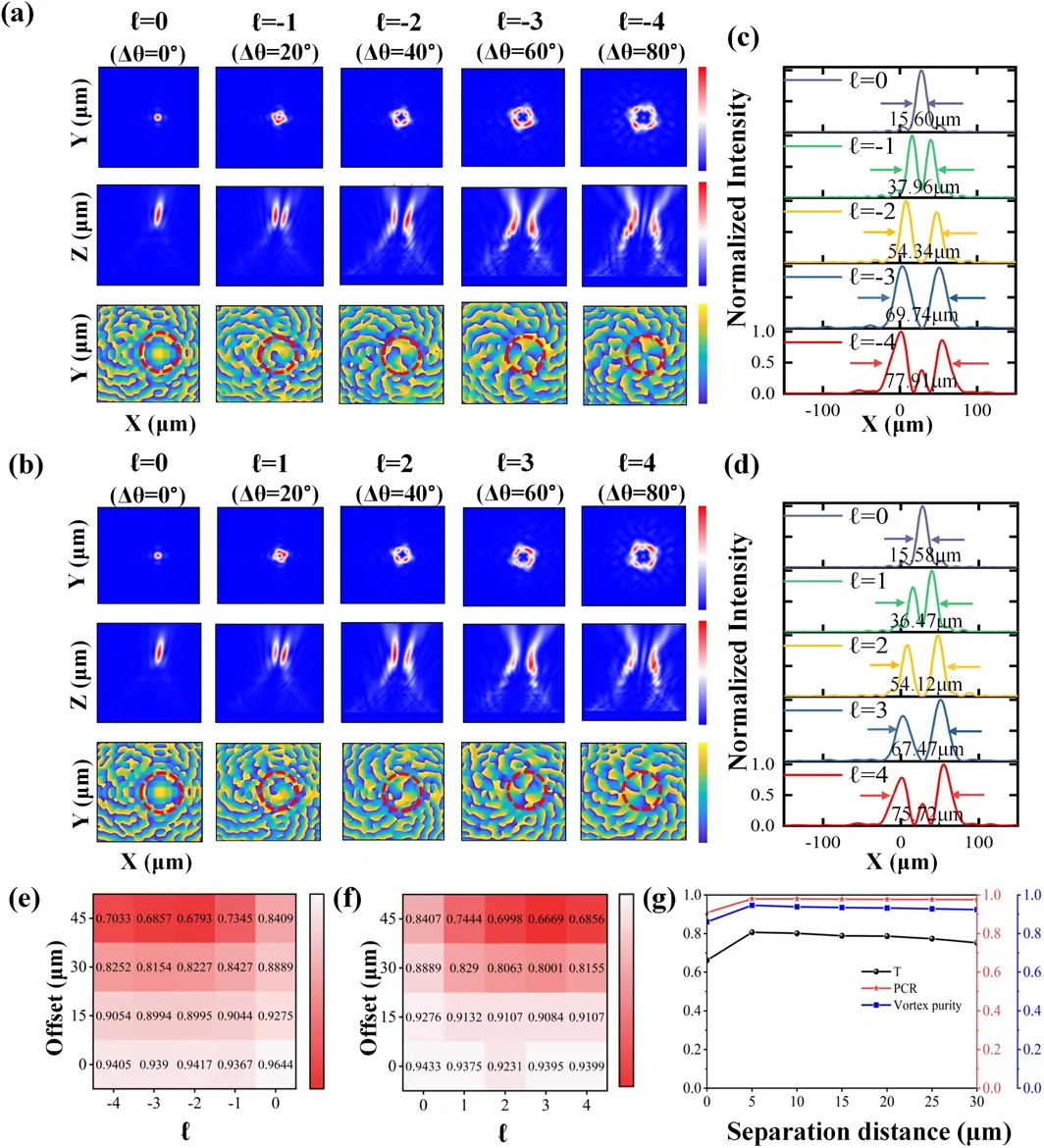
Focal‐plane light field distributions with M_3_ = 0°. (a), (b) XY focal‐plane intensity distributions and corresponding XZ and XY phase distributions under LCP and RCP incidence, respectively. (c), (d) Cross‐sectional intensity profiles and FWHM distributions of conjugate vortex beams with opposite topological charges generated in LCP and RCP channels. (e) Target OAM mode purity under LCP incidence for different topological charges and M_3_‐induced lateral offsets. (f) Target OAM mode purity under RCP incidence for different topological charges and M_3_‐induced lateral offsets. (g) Transmittance, polarization conversion efficiency, and target OAM mode purity versus the axial spacing between the metasurfaces.

## 3 Results and Discussion

We first designed and characterized a two‐layer coaxial moiré metasurface system (M_1_ and M_2_) to demonstrate the feasibility of dynamically manipulating vortex and vector beams based on the moiré effect. This configuration directly superimposes the focusing phase on the discrete moiré phase distribution of the second metasurface M_2_.

Figure 4 illustrates the focal plane light field response of this two‐layer device under different polarization excitations. Figure 4a,b illustrate the evolution of the focal‐plane field under left circularly polarized (LCP) and right circularly polarized (RCP) incidence as the relative rotation angle between metasurfaces M_1_ and M_2_ increases from 0° to 80° in 20° increments. According to the discrete moiré mapping employed here, each 20° increase in the relative rotation angle raises the absolute topological charge of the output beam by one. When the two metasurfaces are aligned with no relative rotation, the output topological charge is *ℓ* = 0, resulting in a solid Gaussian focal spot. As the relative rotation angle increases, the focal‐plane intensity transforms into an annular vortex distribution with a central dark core, and the absolute topological charge increases from 0 to 4. The vortex phases generated under LCP and RCP incidence exhibit opposite handedness, indicating that the two circular polarization channels carry topological charges of equal magnitude but opposite sign.

**FIGURE 4:**
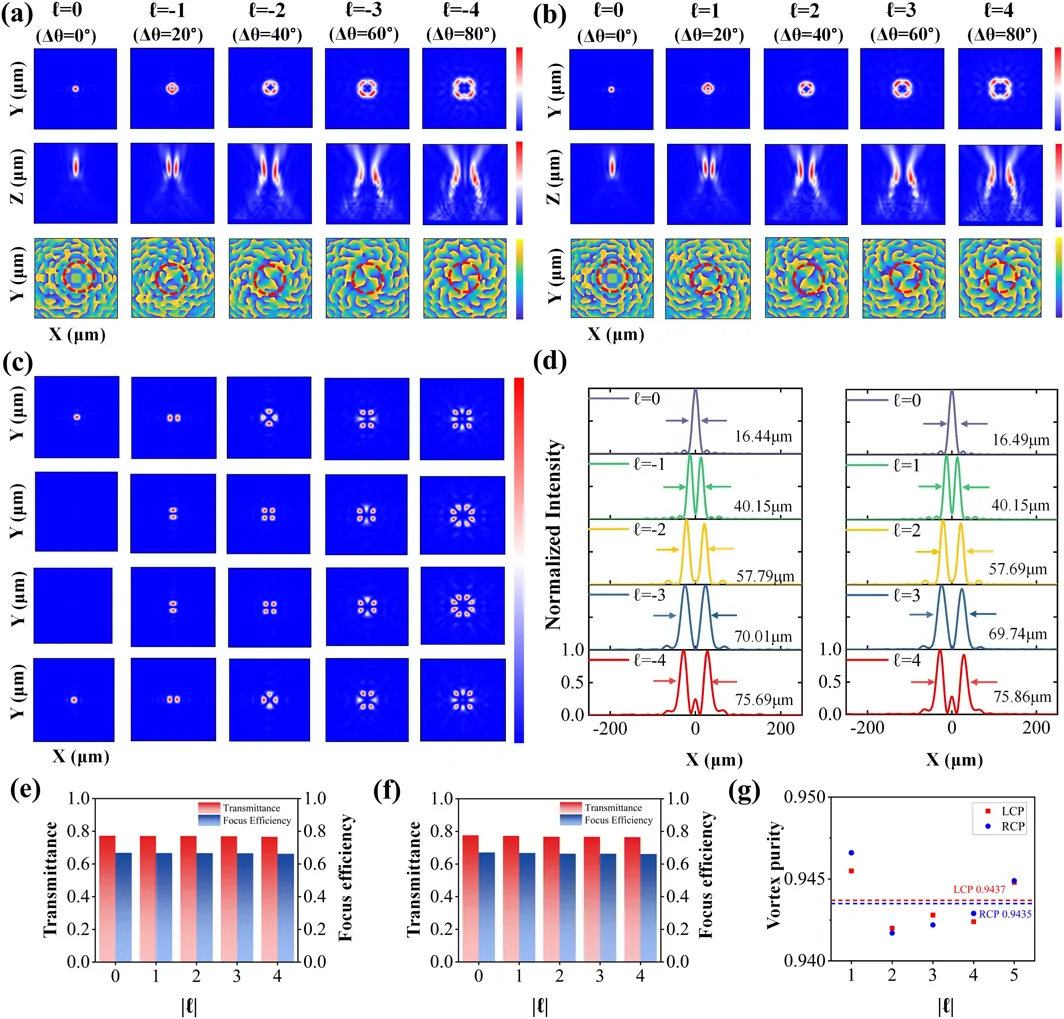
Light field characteristics of the double‐layer metasurface system under different polarization states. (a), (b) XY focal‐plane intensity distributions and corresponding XZ and XY phase distributions under LCP and RCP excitation, respectively. (c) Focal‐plane distributions of polarization component intensities, sequentially presenting the x and y polarization components under x‐polarized incidence and those under y‐polarized incidence. (d) Cross‐sectional intensity profiles and FWHM distributions of vortex beams with opposite topological charges generated in orthogonal circular polarization channels. (e) Transmittance and focusing efficiency under LCP incidence. (f) Transmittance and focusing efficiency under RCP incidence. (g) Purity of the target OAM modes at different relative rotation angles under LCP and RCP incidence.

Figure 4c shows the focusing response of the device when linearly polarized light is incident along the x‐ and y‐directions. When the intensities of the X and Y polarization components are independently extracted using a polarizer, twopetal interference patterns are observed. This petal‐like splitting of the polarization component intensities demonstrates the successful synthesis of a vector beam with a tunable polarization order. This is a characteristic physical manifestation of the coherent superposition of circularly polarized beams with positive and negative topological charges. Figure 4(d) shows the one‐dimensional normalized intensity distribution curves for the cross‐section at different topological charge numbers (from |*ℓ*| = 0 to|*ℓ*| = 4) when LCP and RCP light are incident. A pronounced zero‐intensity point consistently appears at the core. Simultaneously, the full width at half maximum (FWHM) between the two primary intensity peaks increases monotonically as the absolute value of the topological charge increases. The FWHM expands steadily from 16.44 to 75.69 μm under LCP incidence and from 16.49 to 75.86 μm under RCP incidence. Figure 4e,f present the transmittance and focusing efficiency of the cascaded metasurface system under left circularly polarized (LCP) and right circularly polarized (RCP) incidence, respectively. The average transmittances are 77.00% and 76.90%, whereas the average focusing efficiencies are 66.60% and 66.57% for LCP and RCP incidence, respectively. Figure 4g further illustrates the target orbital angular momentum (OAM) mode purity at various relative rotation angles between metasurfaces M_1_ and M_2_. The mean mode purities are 94.37% and 94.35% under LCP and RCP incidence, respectively, demonstrating that high and stable mode quality is maintained during topological mode tuning.

A third‐order metasurface M_3_ was incorporated into the system to enable variable control over the beam's focal position. Initially, we independently evaluated the dynamic tuning performance of the first two cascaded metasurfaces M_1_ and M_2_ for the topological charge of vortex beams. Numerical simulations of the system's near‐field electromagnetic response were performed under various relative rotation angles Δθ, with M_3_'s rotation angle *θ*_3_ fixed at 0°. For clarity, Figure 3 presents only representative low‐order results for |*ℓ*| = 0–4. The complete rotation range, higher‐order mode performance, and rotation‐angle tolerance analysis are provided in Supporting Information S1: Figure S1. Figure 3 illustrates the changes in the light field at the focal plane when LCP and RCP light are incident. The lateral X–Y focal plane intensity distributions shown in Figure 3a,b demonstrate how the focused spot gradually transforms into a characteristic vortex intensity ring as it expands. Further analysis of the XY focal‐plane phase distribution reveals that the wavefront produces highly regular spiral phase discontinuities, corresponding to the phase singularity marked by the yellow dashed circle in the image as Δθ increases. The system effectively generates conjugate topological charges of equal magnitude but opposite signs in the two orthogonal spin channels, as demonstrated by the phase responses in Figure 3a,b. Figure 3c,d present the one‐dimensional normalized intensity distribution curves extracted from the center of the light spot. The zero intensity at the center of the curve further confirms the presence of a phase singularity. Furthermore, when LCP and RCP are incident, the FWHM varies from 15.60 to 77.91 μm and from 15.58 to 75.52 μm, respectively, as the absolute value of the increases from 0 to 4. Figure 3e,f illustrate the target OAM mode purity under LCP and RCP incidence as functions of the topological charge and lateral offset d_offset_. When *d*_offset_ ≤ 30 μm, the target mode purity generally remains above 80%, indicating that lateral focal‐position control has only a limited effect on the OAM mode. As the offset increases further, off‐axis aberrations, finite‐aperture effects, and mode crosstalk gradually reduce the mode purity, although the target mode remains dominant.

To evaluate the cross‐influence between polarization‐order control and focal‐position control, the relative rotation angle of M_1_ and M_2_ was varied while the rotation angle of M_3_ and the designed offset of 30 μm were kept fixed. The focal‐centroid positions under left circularly polarized (LCP) and right circularly polarized (RCP) incidence were then extracted. As shown in Figure 5c, the maximum focal drifts under LCP and RCP incidence are 1.73 and 4.33 μm, respectively, corresponding to only 1.75% and 4.40% of the approximately 98 μm effective spot diameter. These results indicate that the relative rotation of M_1_ and M_2_ primarily controls the topological charge and polarization order, exerting only a minor effect on the focal position. Combined with the results obtained by fixing M_1_ and M_2_ while rotating M_3_—where the polarization order and orbital angular momentum (OAM) mode purity remain nearly stable—the cascaded system can be considered to provide approximate functional decoupling between polarization order and two‐dimensional focal position over the investigated parameter range.

**FIGURE 5:**
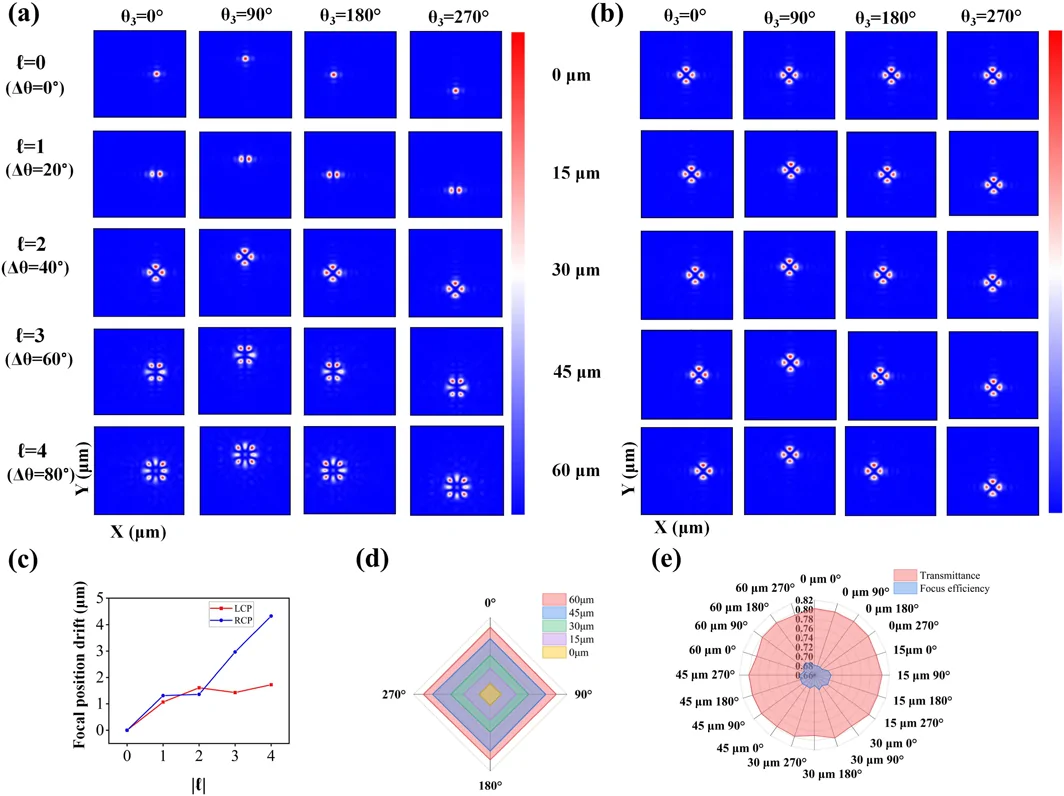
Optical field characteristics and efficiency analysis of spatially tunable vector beams generated by the three‐layer cascaded system. (a) Focal‐plane intensity distributions under different rotation combinations of M_1_, M_2_, and M_3_ with x‐polarized incidence. (b) Off‐axis focal‐plane optical fields at various M_3_ rotation angles and lateral offsets when the relative rotation angle of M_2_ is fixed at 40°. (c) Focal position versus the relative rotation angle Δθ under LCP and RCP incidence for *d*_offset_ = 30 μm and *θ*_3_ = 0°. (d) Actual two‐dimensional spatial translation trajectory of the focal spot with lateral d_offset_ with varying M_3_ rotation angles. (e) Variations of the overall transmittance and absolute focusing efficiency with spatial offset and M_3_ rotation angle.

Building on the established continuous adjustability of the topological charge, Figure 6 further demonstrates the decoupling and translation capabilities of the third‐layer metasurface M_3_ with respect to the beam's lateral spatial position. We fixed the relative rotation angle between M_1_ and M_2_ at Δθ = 40° with a designed lateral offset *d*_offset_ = 30 μm and adjusted the rotation angle of M_3_ to 0°, 90°, 180°, and 270°, respectively. Regardless of whether the incident light is LCP (Figure 6a) or RCP (Figure 6b), the vortex beam spot exhibits an off‐axis circular translation across all four quadrants of the focal plane when the M_3_ mirror undergoes a 90° mechanical rotation. This behavior is evident in the XY focal plane intensity distributions shown in Figure 6a,b.

**FIGURE 6:**
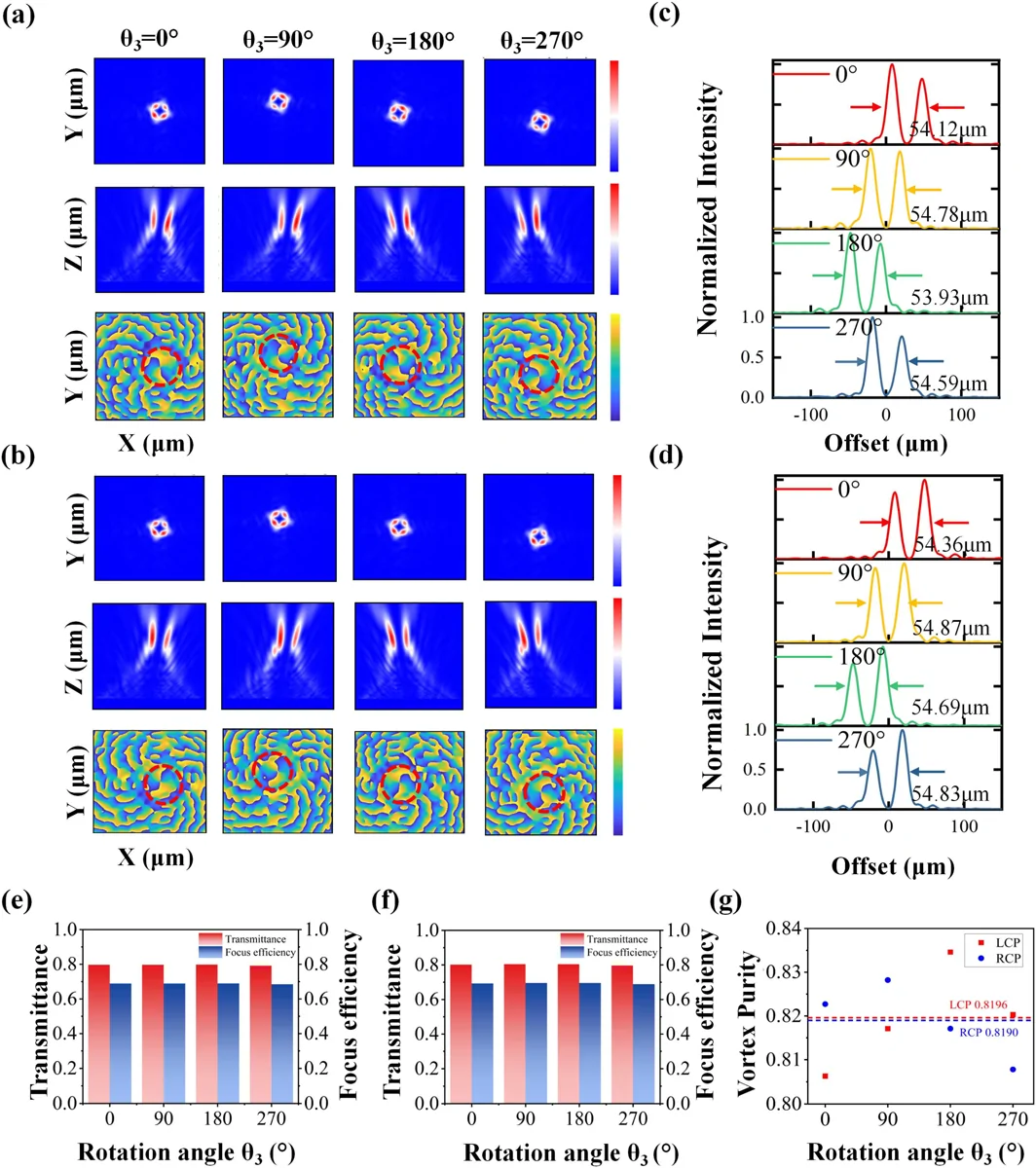
Focal‐plane light field distributions with different M_3_ rotation angles at a fixed relative rotation angle of Δθ = 40°. (a), (b) XY focal‐plane intensity distributions and corresponding XZ and XY phase distributions under LCP and RCP incidence, respectively. (c), (d) Cross‐sectional intensity profiles and FWHM distributions of conjugate vortex beams with opposite topological charges generated in LCP and RCP channels under various M_3_ rotation angles. (e) Transmittance and focusing efficiency under LCP incidence. (f) Transmittance and focusing efficiency under RCP incidence. (g) Target OAM mode purity under LCP and RCP incidence at different M_3_ rotation angles *θ*_3_.

The beam precisely targets the predetermined offset coordinates in the XZ cross‐section, demonstrating off‐axis focusing behavior. Additionally, the internal spiral phase structure undergoes a spatial displacement synchronized with the light intensity envelope, as indicated by the yellow dashed circles in the XY phase map. This indicates that the polarization‐insensitive propagation phase of M_3_ applies approximately the same spatial control to the two circular‐polarization channels. Figure 6c,d display the one‐dimensional normalized intensity distribution of the light spot along the offset axis. The bimodal structure remains highly consistent across the four rotation angles, with the peak centers precisely shifted to spatial offset distances of equal magnitude but opposite sign. This confirms that the system exhibits excellent spatial positioning and routing capabilities. Figure 6e,f further illustrate the transmittance and focusing efficiency of the cascaded system at different *θ*_3_ angles. Both metrics exhibit only slight variations with the rotation angle of M_3_. The mean transmittances under left circularly polarized (LCP) and right circularly polarized (RCP) incidence are 79.29% and 79.96%, respectively, whereas the mean focusing efficiencies are 68.57% and 68.63%, respectively. To assess the effect of lateral focal‐position control on the orbital angular momentum (OAM) mode, Figure 6g presents the target OAM mode purity of the second‐order vector beam at various M_3_ rotation angles. The mean mode purities in the LCP and RCP channels are 0.8196 and 0.8190, respectively. These results demonstrate that, at a lateral offset (d_offset_) of 30 μm, rotation of M_3_ shifts the lateral focal position while largely preserving transmittance, focusing efficiency, and the target OAM mode.

To comprehensively evaluate the beam control performance and operational robustness of the three‐layer cascade metasurface system, this study systematically tested the output optical field characteristics of M_3_ under various rotation angles and preset offset conditions. As shown in Figure 5a, when the offset is set to 60 microns, under x‐linearly polarized light excitation, we systematically studied the near‐field optical field distributions of M_1_, M_2_, and M_3_ under different rotation angle combinations. The structural integrity of the vector beam and the polarization topological states were very well preserved.

As shown in Figure 5b, with the relative rotation angle of M_1_ and M_2_ fixed at 40°, the beam offset was gradually increased from 0 to 60 μm, while setting the rotation angle of M_3_ to 0°, 90°, 180°, and 270°, respectively, and the near‐field intensity distributions under each condition were collected and analyzed. The results show that even under the extreme off‐axis offset condition of 60 μm, the beam's characteristic four‐lobed intensity distribution maintained good symmetry and very high imaging contrast, with no significant distortion or aberration in the internal morphology of the vector beam. Figure 5d presents the actual focal spatial mapping distributions corresponding to multiple parameter sets. Polar coordinate analysis indicates that the focal coordinates corresponding to different offsets exhibit a regular geometric nested structure. These results demonstrate that the actual beam translation trajectory has excellent linearity and fidelity with the theoretical spatial mapping transformation, confirming the feasibility and reliability of achieving high‐precision two‐dimensional coordinate beam addressing through single‐axis mechanical rotation. Figure 5e presents the transmittance and absolute focusing efficiency of the system for different combinations of lateral offset and M_3_ rotation angle. Here, the absolute focusing efficiency is strictly defined as follows: On the focal plane, integrate the light intensity over the spot area where the vortex light intensity decays from its peak value to 1/e^2^ of the original value. Calculate the ratio of the energy within this effective area to the total transmitted light energy, and finally multiply this value by the overall absolute transmittance of the system.

Over lateral offsets ranging from 0 to 60 μm, the transmittance and absolute focusing efficiency remain above 77% and 67%, respectively. Increasing the offset slightly reduces the focusing efficiency due to off‐axis aberrations and finite‐aperture effects, whereas M_3_ rotation has minimal influence.

Figure 7a,b show the Stokes parameters of the vector beams generated by the system under X and Y polarization excitation, respectively, when *θ*_3_ is 180°, and the relative rotation angle Δθ of the first two layers is varied (*ℓ* = 0, 1, 2, 3, 4). The S0 parameter represents the total light intensity distribution, whereas the S1 and S2 parameters characterize the intensity differences between horizontal/vertical linear polarization and diagonal polarization, respectively. The S3 parameter describes the circular polarization component. As shown in the figures, S1 and S2 exhibit alternating symmetrical multi‐lobe patterns. Mathematically, the angular distributions of S1 and S2 are proportional to cos(2ℓα) and sin(2ℓα), respectively. Therefore, both exhibit 4ℓ alternating positive and negative polarization lobes. When ℓ = 1, four petals appear, and when ℓ = 2, eight petals are observed, as illustrated. This pattern provides clear and convincing evidence that the polarization order of the beam is accurately controlled by the relative rotation of M_1_ and M_2_, consistent with our theoretical derivations. The resulting vector beam maintains a pure linear polarization state at every local position across the cross‐section, preventing elliptical polarization degradation caused by phase mismatch. This is confirmed by the figure, where the S3 distribution is nearly zero for all orders.

**FIGURE 7:**
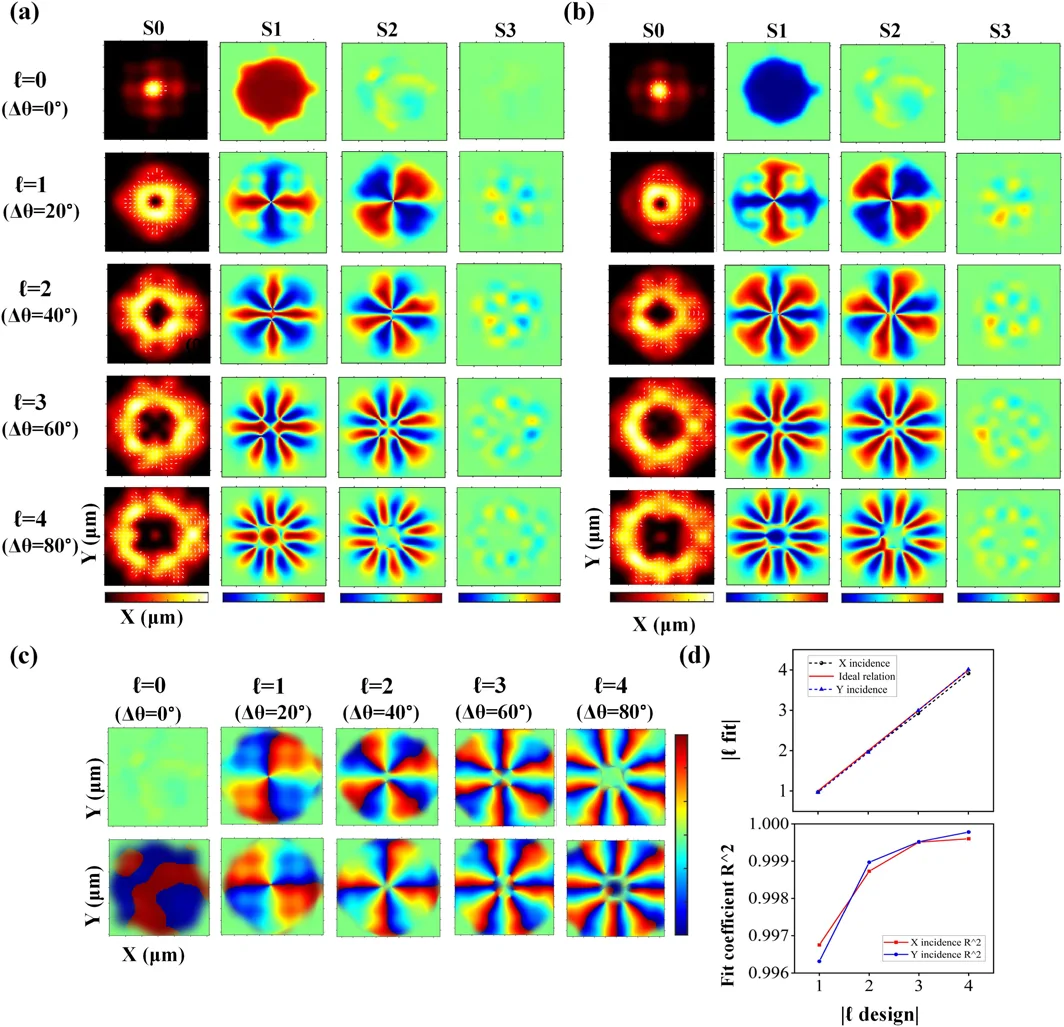
Polarization topological characteristics and Stokes parameter analysis of vector beams based on Moiré metasurfaces. (a), (b) Evolution of the spatial distributions of Stokes parameters (S0, S1, S2, S3) during the dynamic modulation of vector beam polarization orders under x‐ and y‐linear polarized excitation, respectively. (c) Local polarization–axis distributions for different designed orders under x‐and y‐polarized incidence. (d) Polarization order |ℓ_fit_| obtained by fitting the local polarization–axis angle and the corresponding coefficients of determination.

To quantify the local polarization orientation, the polarization–axis angle *ψ* is calculated using the Stokes parameters:

$$\psi =\frac{1}{2}\arctan\left(\frac{S_{2}}{S_{1}}\right).$$

Because the linear polarization axis is unoriented, *ψ* = *ψ* + π. Only regions that meet the specified intensity and degree‐of‐linear‐polarization thresholds are included to suppress numerical errors in low‐intensity areas. Figure 7c shows the polarization–axis distributions for *ℓ* = 1–4 under x‐ and y‐polarized illumination. As |*ℓ*| increases, the azimuthal winding frequency of the polarization axis increases, consistent with the designed order. The polarization–axis angle within the effective annular region is further fitted by the following equation:

$$\psi \left(\varphi \right)=l_{fit}\varphi +\psi_{0},$$

where φ is the azimuthal angle, $l_{\text{fit}}$ is the fitted order, and $\psi_{0}$ is the initial polarization orientation. As shown in Figure 7d, the fitted $|l_{\text{fit}}|$ agrees well with the designed values for both incident polarizations, with all $R^{2}$ values exceeding 0.996. These results confirm that the cascaded metasurface preserves the prescribed polarization–axis topology with high fidelity.

Experimental rotational moiré metasurfaces have typically utilized a fixed–rotating configuration, where one layer remains stationary while the other is mounted on either a manual existing rotating moiré metasurface experiments commonly use an assembly in which one layer is fixed and the other is rotated: One metasurface is fixed, whereas the other is mounted on a manual [34] or motorized rotation stage [44], maintaining precise in‐plane alignment. Multilayer metasurface systems have also employed face‐to‐face chip alignment, lithographic alignment marks, microscale spacers, and fixation outside the clear aperture to control interlayer assembly and spacing [42, 43]. Based on these methods, the proposed three‐layer system could utilize a conceptual coaxial assembly. M_1_ would be fixed to a reference mount, whereas M_2_ and M_3_ would be installed in two independently driven hollow rotation mechanisms sharing a common mechanical reference. The relative rotation between M_1_ and M_2_ would tune the topological charge and polarization order, whereas the rotation of M_3_ would control the lateral focal position. The 10 μm interlayer gaps could be defined by either three equal‐height microspacers or an annular spacer placed outside the clear aperture. Layer centers and initial angles could be calibrated using lithographic marks and microscope imaging, whereas substrate parallelism and interlayer spacing could be verified through interferometric or confocal measurements. This assembly represents a feasible implementation route based on existing methods but has not been experimentally validated in the present work.

Within this scheme, both the polarization order and the lateral focal position of the vector beam can be adjusted on the same platform. In multi‐target optical manipulation, M_3_ steers the focus, while the relative rotation of M_1_ and M_2_ tunes the polarization order and orbital angular momentum (OAM) mode. In mode‐routing or mode‐multiplexing systems, the polarization/OAM order can serve as a modal channel, and the focal coordinate can function as a spatial output port. This combined mode–position control may also benefit multi‐position structured illumination and laser processing.

This study demonstrates the numerical feasibility of the proposed mechanism. However, practical implementation will be influenced by factors such as rotation speed, multilayer alignment, interlayer spacing errors, fabrication deviations, and cascade insertion loss. Consequently, mechanical rotation is better suited for quasi‐static or low‐frequency operation. Future research may integrate MEMS actuation, embedded rotation mechanisms, inverse design, and wide‐field aberration correction to enhance tuning speed, integration, mode purity, and positioning accuracy.

## 4 Conclusion

This study proposes and numerically validates a multidimensional dynamic optical‐field reconstruction scheme based on cascaded moiré metasurfaces. The first two layers generate conjugate vortex beams with opposite topological charges in the left‐ and right‐circularly polarized (LCP and RCP) channels, and their relative rotation adjusts the polarization order of the resulting vector beam under linearly polarized illumination. Rotation of the third layer controls the lateral focal position within the focal plane. Over lateral offsets ranging from 0 to 30 μm, the two control degrees of freedom exhibit limited cross‐influence, whereas the dominant orbital angular momentum (OAM) mode and primary polarization–topology features are preserved during two‐dimensional steering. At larger offsets, off‐axis aberrations reduce mode purity and local polarization consistency, although the target mode remains dominant. The analysis also identifies the main causes of this degradation and suggests possible routes for improvement. This compact platform extends moiré metasurface control from on‐axis focusing to combined mode and spatial‐position tuning, with potential applications in multi‐target optical manipulation, spatial‐mode routing, and laser micromachining.

## Author Contributions

**Siyu Guo:** methodology, writing – original draft, writing – review and editing, resources, conceptualization, investigation, formal analysis, visualization, data curation. **Zhongzhu Liang:** data curation, writing – review and editing, project administration, funding acquisition, supervision, resources. **Zhe Wu:** software. **Xintong Wei:** investigation. **Shijia Zhu:** supervision. **Junying Liu:** formal analysis. **Siyu Liu:** visualization. **Qiaohan Liu:** visualization, project administration. **Xiaoyan Shi:** conceptualization, visualization. **Fuming Yang:** data curation, validation. **Haiyang Xu:** funding acquisition.

## Conflicts of Interest

The authors declare no conflicts of interest.

## Supporting information


Supporting Information S1

## Data Availability

The data that support the findings of this study are available on request from the corresponding author. The data are not publicly available because of privacy or ethical restrictions.
