# Supplementary material for: Dynamic Generation of Spatially Variable Vector Beams Using Cascaded Moiré Metasurfaces
Source: Nanophotonics. 2026 Aug 25;15(17):e70261. doi: 10.1002/nap2.70261 (PMC13672135; doi:10.1002/nap2.70261)
Supplement: Supplementary file 1 [file NAP2-15-e70261-s001.docx]

**Supporting information**

**Dynamic Generation of Spatially Variable Vector Beams Using Cascaded Moiré Metasurfaces**

*Siyu Guo^1^，Zhongzhu Liang^*,1^，Zhe Wu^1^，Xintong Wei^1^，Shijia Zhu^1^，Junying Liu^1^，Siyu Liu^1^，Qiaohan Liu^1^，Xiaoyan Shi^1^，Fuming Yang^1^，Haiyang Xu^*,1^*

*^1^State Key Laboratory of Integrated Optoelectronics and Key Laboratory of ultraviolet Light-Emitting Materials and Technology of Ministry of Education, College of Physics, Northeast Normal University, Changchun 130024, China*

** e-mail: [liangzz@nenu.edu.cn](mailto:liangzz@nenu.edu.cn), hyxu@nenu.edu.cn*

**Table 1: Dimensions of 16 isotropic unit structures**

|  | **L(µm)** |  | **L(µm)** |
| --- | --- | --- | --- |
| **1** | 1.47 | **9** | 2.21 |
| **2** | 1.72 | **10** | 2.27 |
| **3** | 1.82 | **11** | 2.33 |
| **4** | 1.92 | **12** | 2.37 |
| **5** | 2.00 | **13** | 2.63 |
| **6** | 2.05 | **14** | 2.70 |
| **7** | 2.10 | **15** | 2.89 |
| **8** | 2.16 | **16** | 3.12 |

To determine the effective tuning range of M_1_ and M_2_, the relative rotation angle Δθ was varied over the full 0°–360° range. The orbital angular momentum (OAM) spectra and target-mode purities were calculated separately under left circularly polarized (LCP) and right circularly polarized (RCP) illumination. As shown in Figures S1(a) and S1(c), within the principal rotation interval of 0° ≤ Δθ < 180°, the designed topological charge magnitude increases by one integer order for every 20° increment in Δθ. According to this discrete mapping, the highest nominally encoded order is |ℓ| = 8, corresponding to Δθ = 160°.

The nominally accessible order, however, does not represent the upper limit of high-fidelity operation. As |ℓ| increases, the target-mode purity gradually decreases, accompanied by stronger contributions from neighboring OAM modes. At |ℓ| = 5, the target-mode purity is approximately 91.99% for both the LCP and RCP channels. For |ℓ| = 6, the corresponding purities are 86.59% and 87.04%, respectively, with the target mode remaining dominant. A more pronounced reduction occurs at |ℓ| = 7 and 8, where adjacent OAM side modes become increasingly significant. Using a target-mode purity of 75% as the criterion for effective operation, the high-fidelity tuning range extends to Δθ = 120°, corresponding to a maximum reliable topological-charge magnitude of |ℓ| = 6. When Δθ exceeds 180°, the system enters the next rotation period. Simulations indicate that the sign of the generated topological charge reverses relative to the corresponding state below 180°, while its magnitude repeats periodically. Because the helical-phase signs in the LCP and RCP channels are simultaneously reversed, the x-polarized component of the synthesized vector beam also changes accordingly. Representative focal-plane intensity, phase, and x-polarized intensity distributions are included in Fig. S1(a).

The reduction in high-order mode purity results from several factors. First, the transverse size of the vortex beam increases with |ℓ|, making the field more susceptible to truncation by the finite aperture. Second, higher-order helical phases exhibit steeper azimuthal phase gradients, which cannot be fully reconstructed due to the finite unit-cell period and discrete phase sampling. This limitation increases coupling between the target mode and neighboring orbital angular momentum (OAM) orders. Additionally, the accuracy of the local periodic approximation and the independent-cell approximation decreases as the local phase gradient becomes larger. High-order modes are also generally more sensitive to relative rotation errors, lateral misalignment, variations in axial separation, and substrate tilt. Combined with diffraction and residual aberrations during cascaded propagation, these effects redistribute energy from the target mode into adjacent OAM components, ultimately limiting the high-fidelity tuning range.

**Figure S1.** OAM tuning characteristics of the M1–M2 pair over the relative rotation range. (a) Representative focal-plane intensity, phase, and x-polarized component distributions for selected high-order OAM states and for Δθ > 180°. (b) Focal-plane intensity and phase distributions at odd multiples of half the design step, specifically Δθ = 10°, 30°, 50°, 70°, and 90°. (c) Target OAM-mode purity in the LCP and RCP channels over the full relative rotation range. The horizontal dashed line indicates the 75% purity criterion adopted to define the effective operating range.

To examine the response at non-design rotation angles, additional simulations were performed for Δθ = 10°, 30°, 50°, 70°, and 90°, as shown in Fig. S1(b). Since a 20° relative rotation corresponds to a unit change in the designed topological charge, these angles correspond to effective half-integer phase orders of |ℓ_eff_| = 0.5, 1.5, 2.5, 3.5, and 4.5. Unlike the integer-order design states, a half-integer azimuthal phase does not accumulate an integer multiple of 2π after one complete revolution around the optical axis. The simulated fields still exhibit a gradual transition from lower- to higher-order vortex states, with the transverse beam size increasing as |ℓ_eff_| becomes larger. Nevertheless, the azimuthal uniformity and rotational symmetry of the annular intensity distribution deteriorate. The central dark core and ring profile also become increasingly distorted, while distinct phase-discontinuity regions appear in the phase maps, indicating enhanced coupling between adjacent OAM modes. These results confirm that continuous mechanical rotation produces transitional fields between neighboring integer-order states, although their mode purity and vector-beam quality are lower than those obtained at the designed integer-order angles.

**Figure S2**. Sensitivity of the cascaded system to relative rotation errors between M1 and M2. (a) Focal-plane intensity and phase distributions under left circularly polarized (LCP) and right circularly polarized (RCP) illumination as functions of the relative rotation error, δθ. (b) Target orbital angular momentum (OAM) mode purity in the LCP and RCP channels as a function of δθ for a nominal relative rotation angle of Δθ = 40°. (c) Corresponding target mode purity for a nominal relative rotation angle of Δθ = 80°.

To evaluate the system's sensitivity to mechanical rotation errors, two representative states within the high-fidelity operating range were selected, with nominal relative rotation angles of Δθ = 40° and 80°. Angular deviations of δθ = −5°, −3°, −1°, 1°, 3°, and 5° were introduced around each nominal state, and the target OAM-mode purities were calculated for both LCP and RCP illumination. As shown in Figures S2(b) and S2(c), the mode purity exhibits only minor fluctuations around the nominal states. Throughout the investigated range of |δθ| ≤ 5°, the reduction from the highest purity obtained within each angular scan remains below 5%, and the target OAM mode continues to dominate. These results indicate that the system maintains reasonable tolerance to small relative rotation errors and that angular deviations within the tested ±5° range do not cause substantial degradation of the target OAM state.
